# Supplementary material for: TRIM7 Restricts Coxsackievirus and Norovirus Infection by Detecting the C-Terminal Glutamine Generated by 3C Protease Processing
Source: Viruses. 2022 Jul 23;14(8):1610. doi: 10.3390/v14081610 (PMC9394474; doi:10.3390/v14081610)
Supplement: Supplementary file 1 [file viruses-14-01610-s001.zip › viruses-1814341-supplementary.pdf]

Figure S1. ITC traces for GYG protein and main peptides

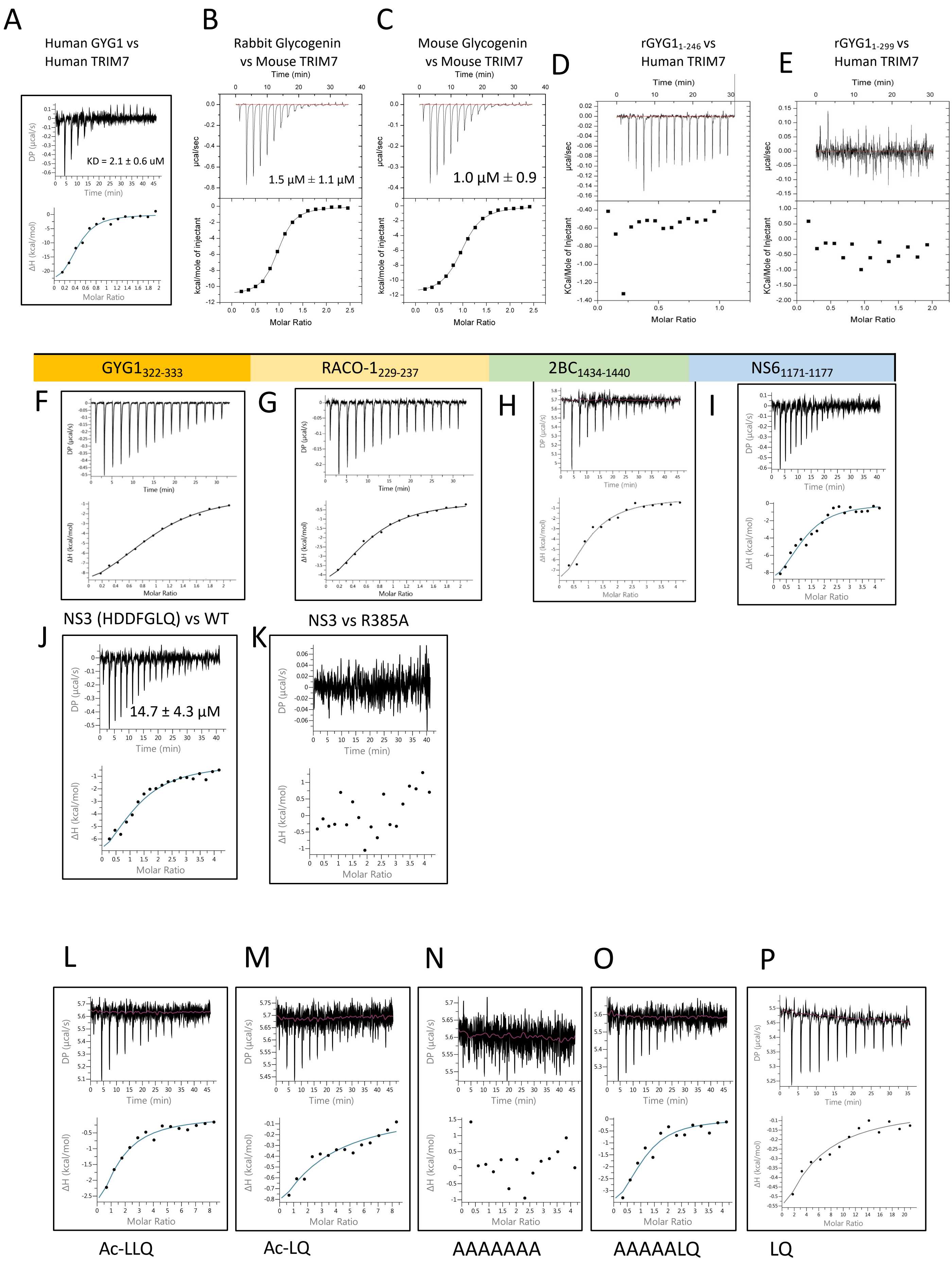

Figure S2. Crystal structures of TRIM7-peptide complexes

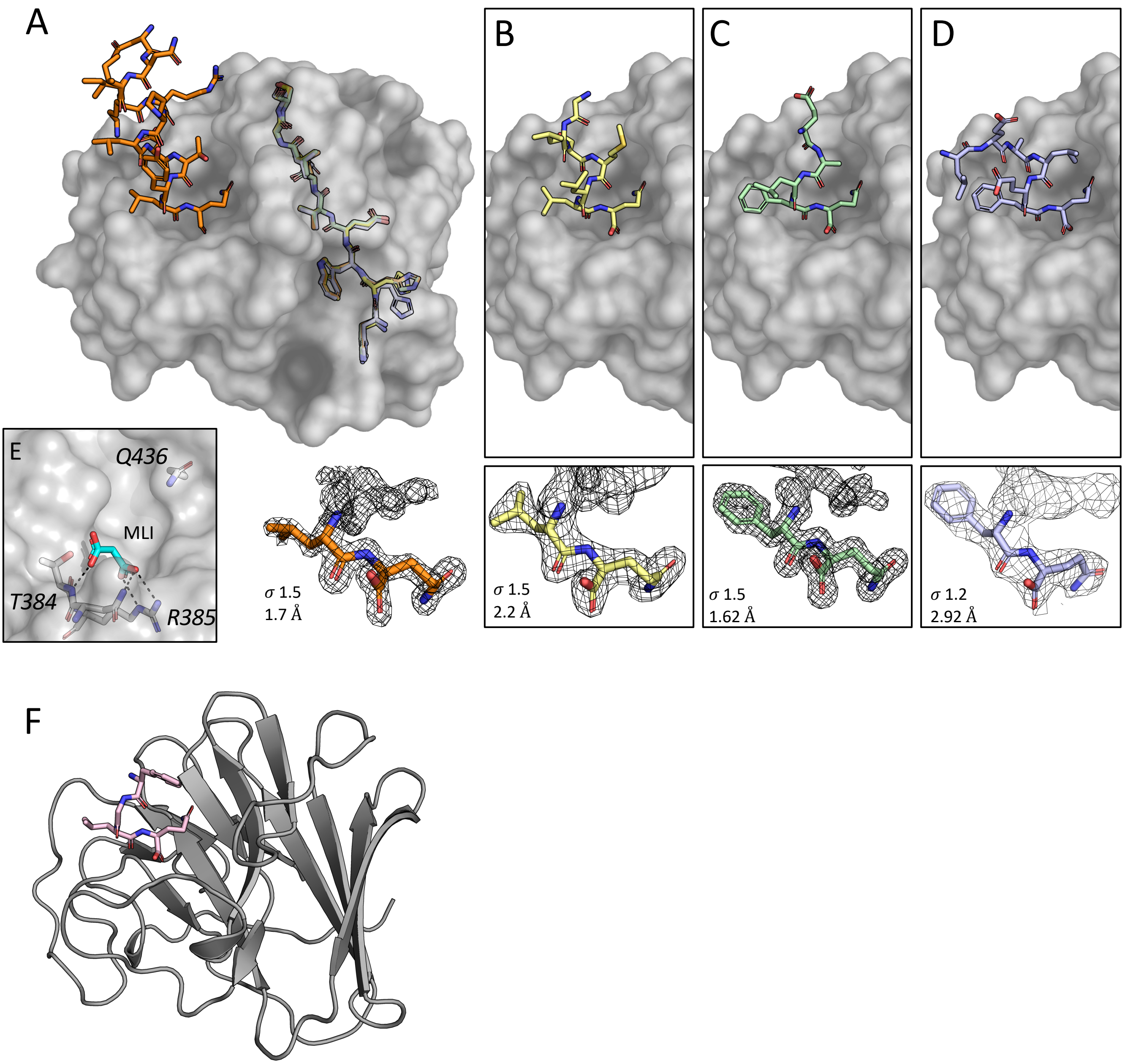

Figure S3 ITC traces for mutant TRIM7 proteins and peptides

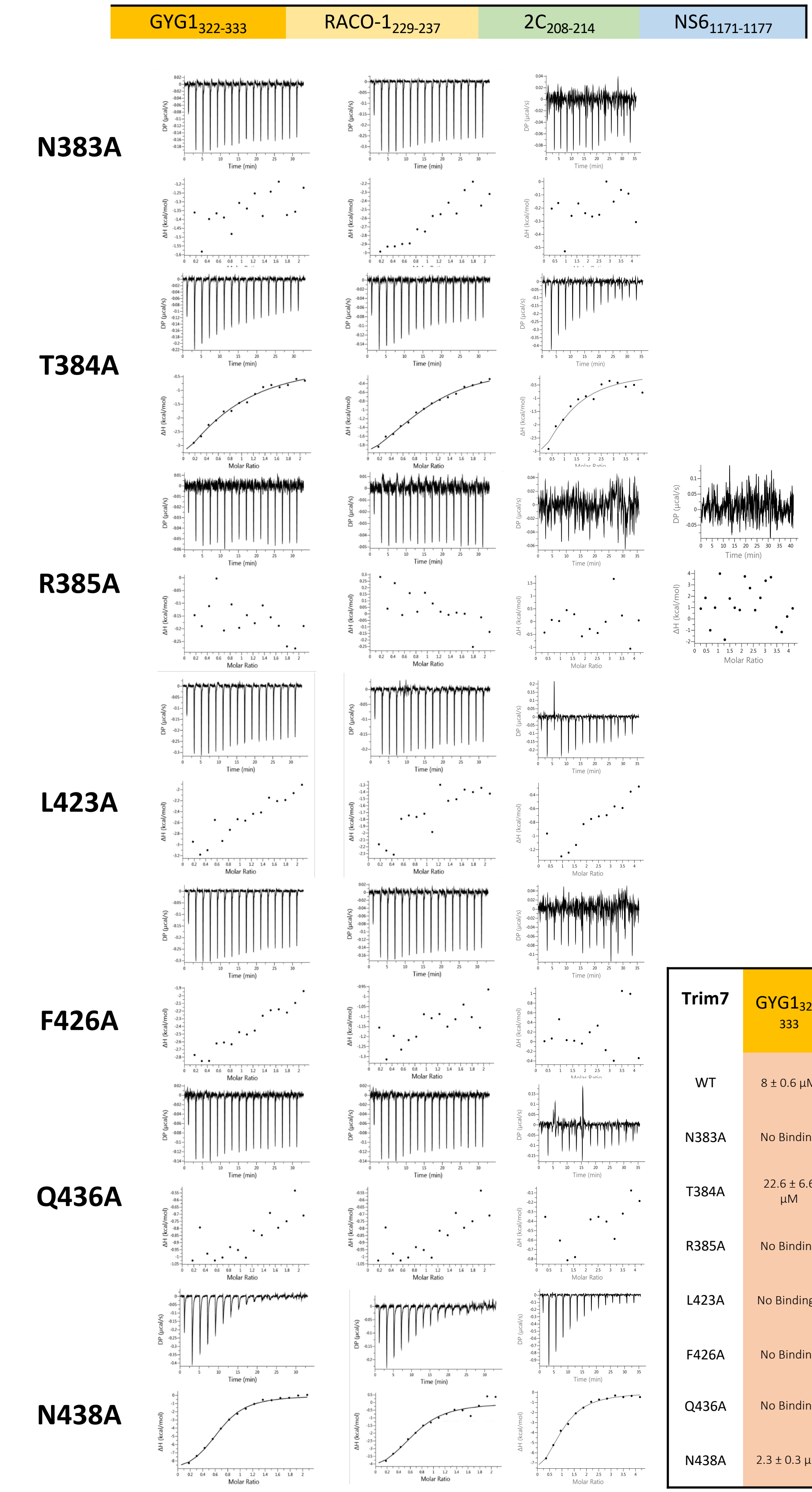

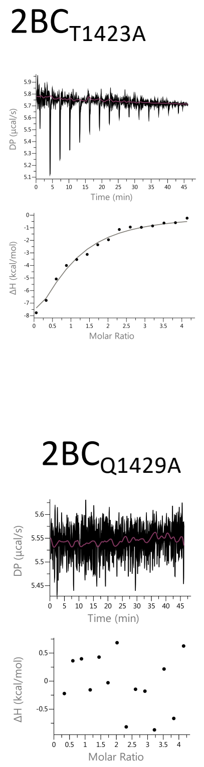

Trim7	$GYG1_{322-333}$	$RACO\text{-}1_{229-237}$	$2BC_{1423-1429}$	$NS6_{1171-1177}$
WT	8 ± 0.6 µM	7.9 ± 1.0 µM	11.2 ± 4.2 µM	8.7 ± 3.2 µM
N383A	No Binding	No Binding	No binding	NT
T384A	22.6 ± 6.6 µM	12.3 ± 2.0 µM	21.2 ± 5.84 µM	NT
R385A	No Binding	No Binding	No binding	No binding
L423A	No Binding*	No Binding*	No binding*	NT
F426A	No Binding	No Binding	No binding	NT
Q436A	No Binding	No Binding	No binding	NT
N438A	2.3 ± 0.3 µM	3.9 µM ± 1.8 µM	7.82 ± 1.18 µM	NT

Figure S4. GYG isoforms

A

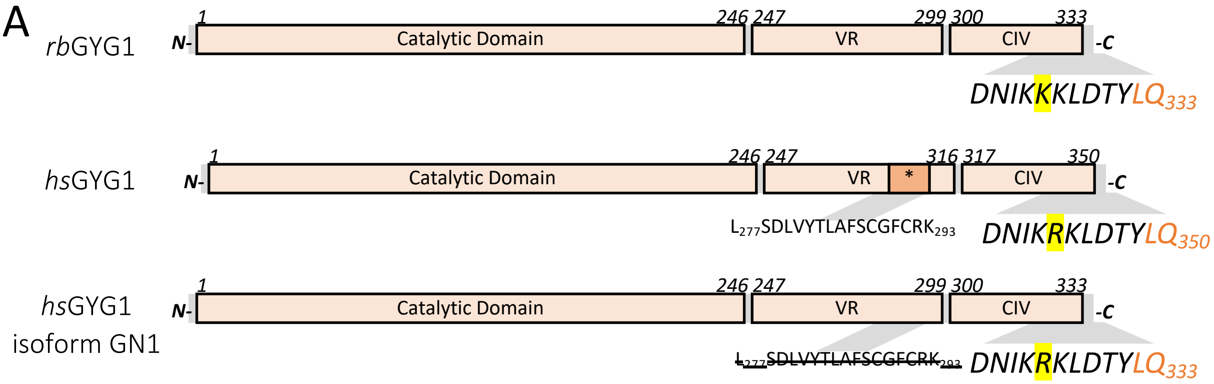

Figure S5. GYG1 colocalizes to TRIM7 cytoplasmic bodies

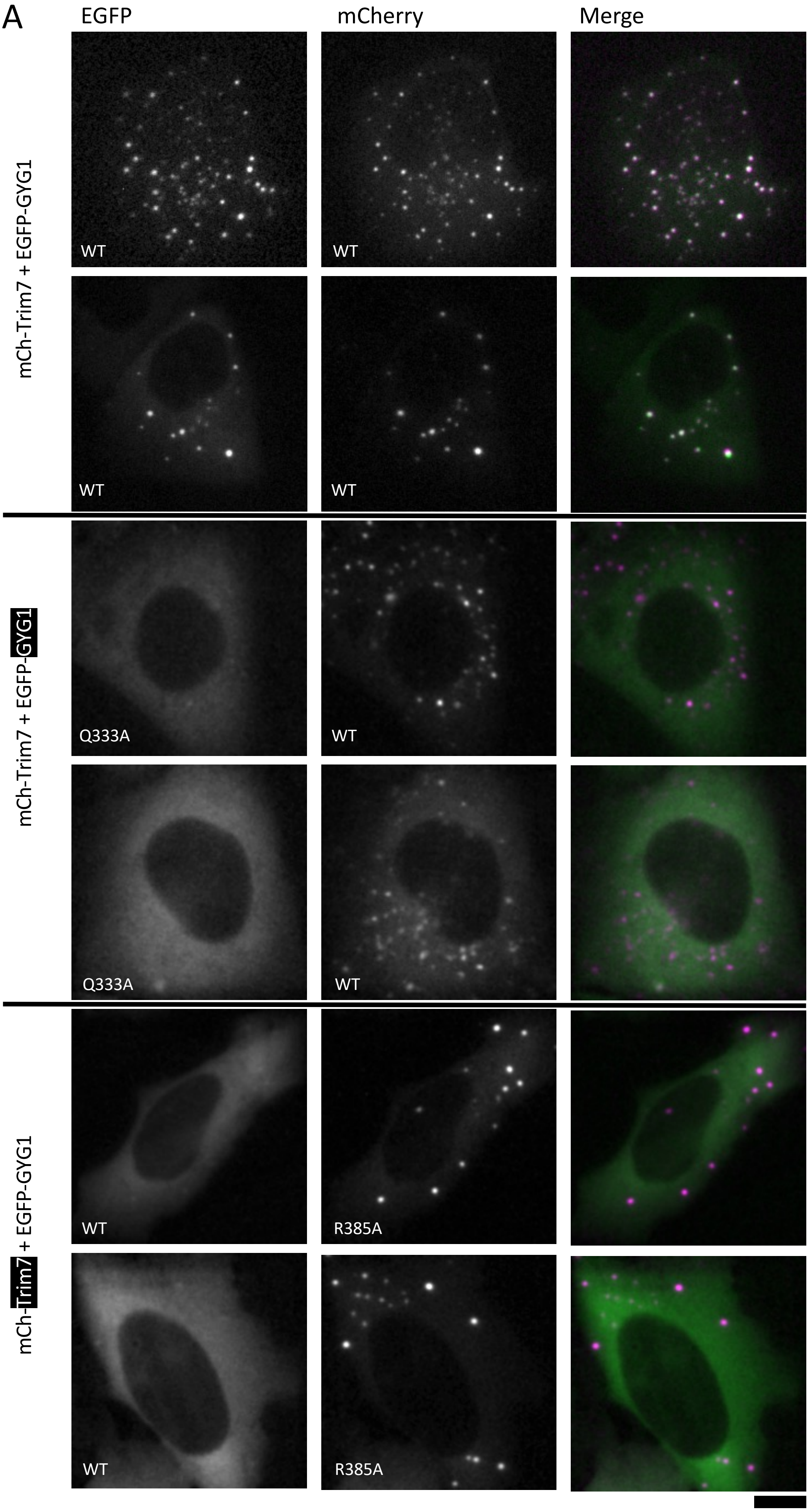

Figure S6. Stable cell line validation.

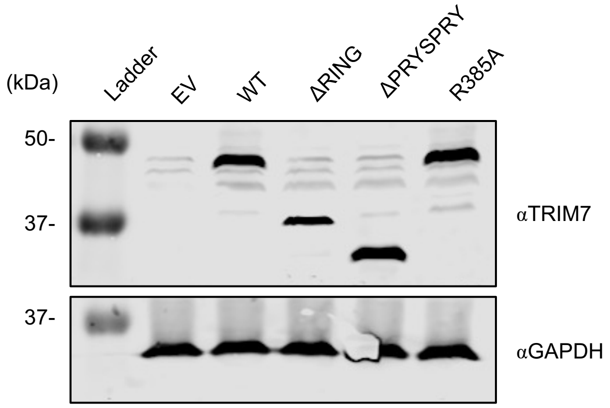

Figure S7. SARS-CoV-2 infection is not restricted by TRIM7

A

Protein	C-term
NSP4	'LQ'
NSP5	'FQ'
NSP6	'VQ'
NSP7	'LQ'
NSP8	'LQ'
NSP9	'LQ'
NSP10	'LQ'
NSP11	
NSP12	'LQ'
NSP13	'LQ'
NSP14	'LQ'
NSP15	'LQ'

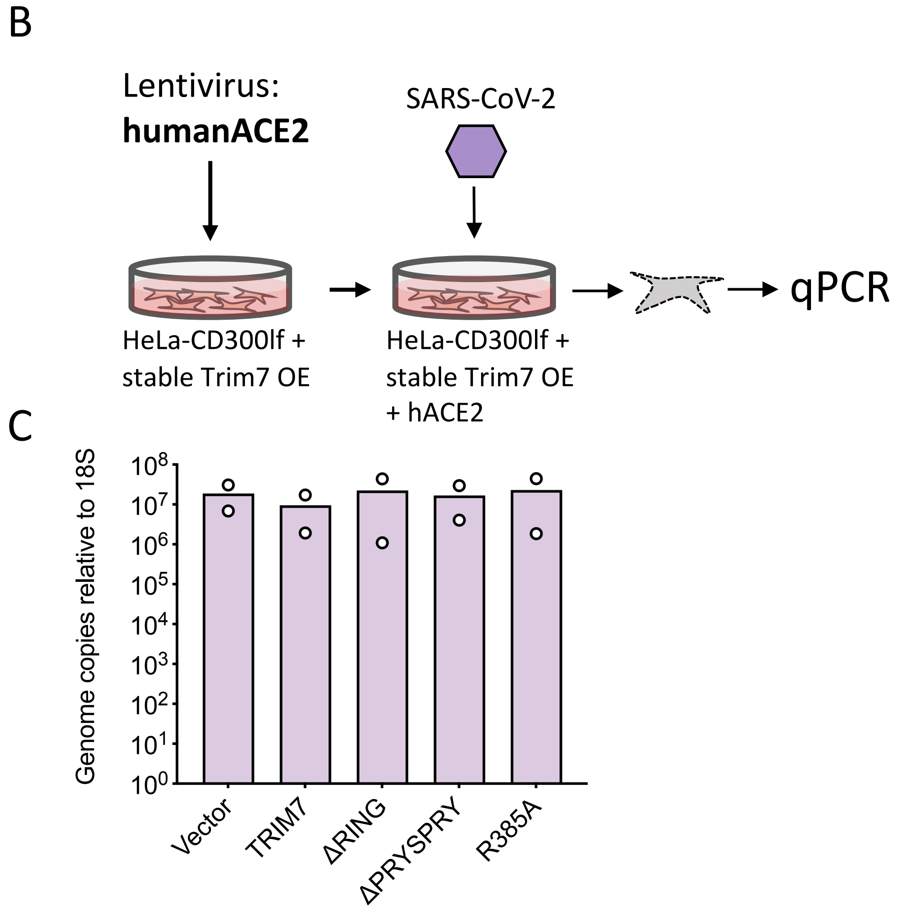

Figure S8. Phylogenetic trees for GYG1 and TRIM7

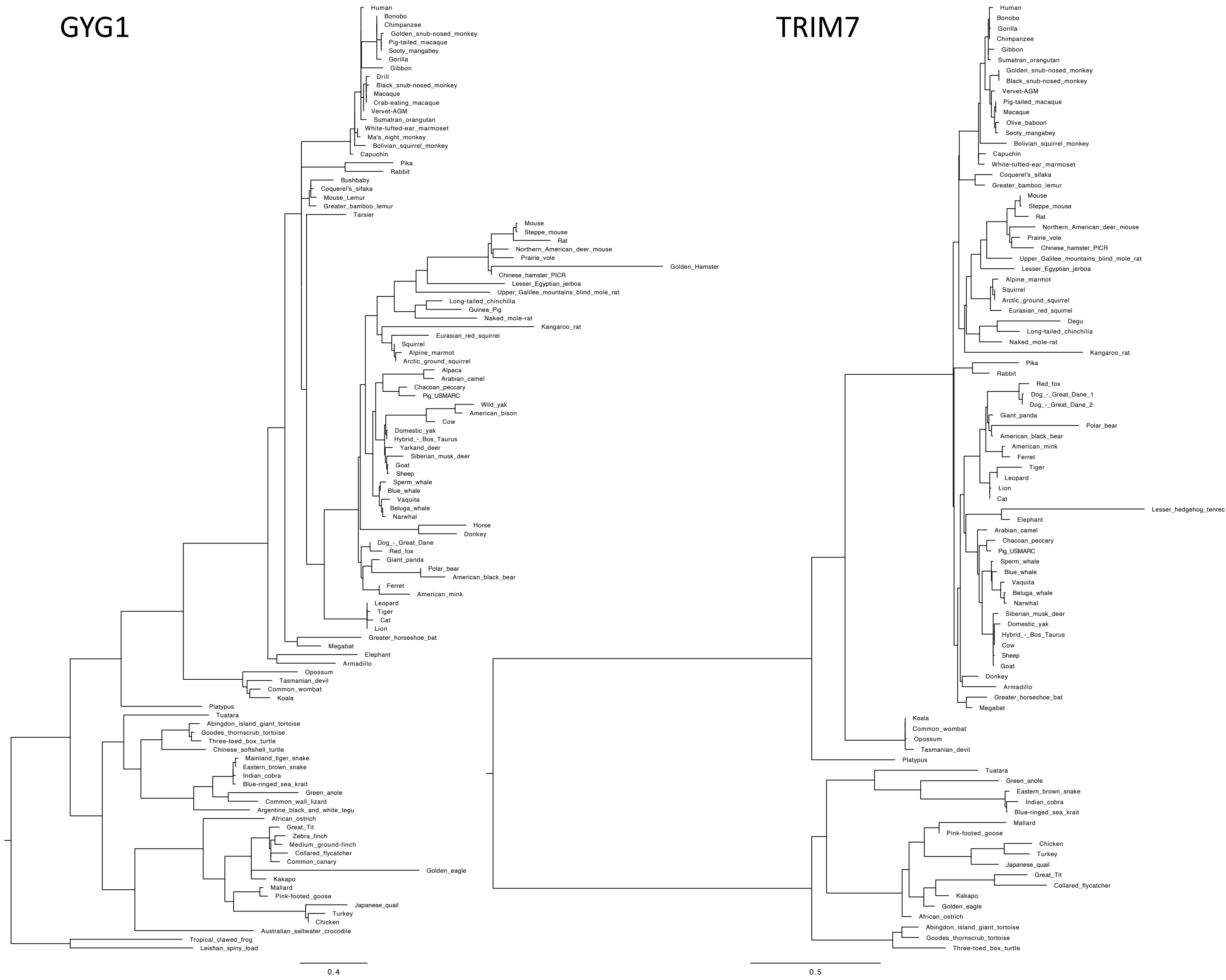

Figure S9. Tissue expression of TRIM7 and associated proteins

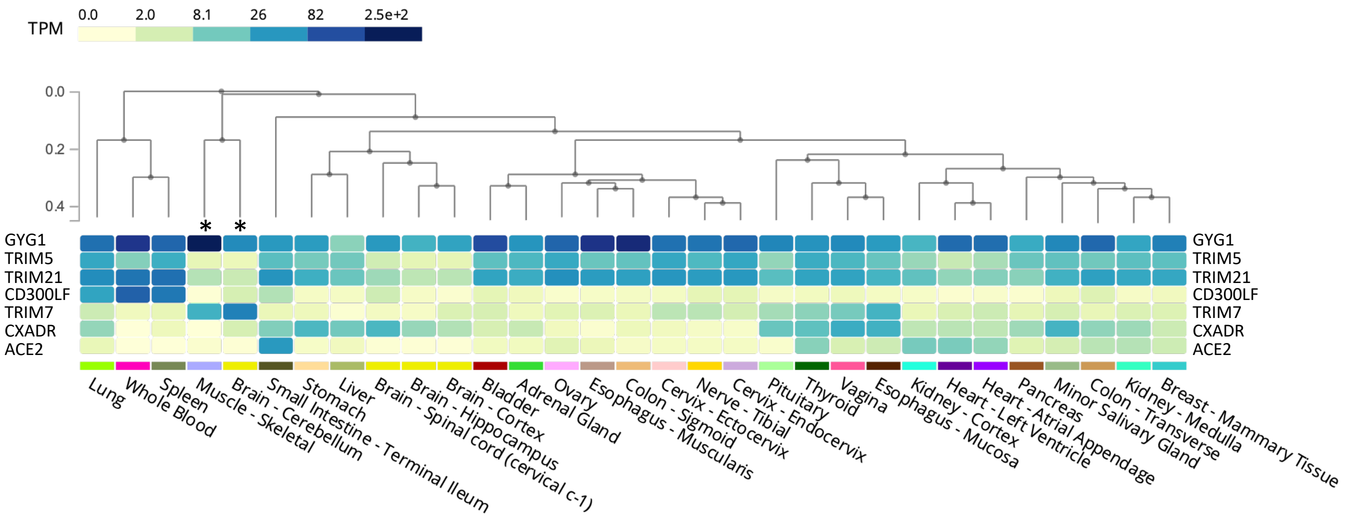

Table S1 Data collection and refinement statistics

	7OVX (TRIM7: GYG1$_{322-333}$)	7OW2 (TRIM7: RACO-1$_{229-235}$)	8A5L (TRIM7:2C$_{208-214}$)	8A5M (TRIM7:MNV1-NS6$_{1171-1177}$)	8A8X (TRIM7:MNV1-NS3$_{699-705}$)
**Data collection**					
Space group	P212121	P61	P65	P1211	P1211
Cell dimensions					
a, *b*, *c* (Å)	37.63, 54.23, 81.40	108.12, 108.12, 137.14	79.89, 79.89, 53.2	40.92, 112.97, 53.04	51.25, 53.67, 73.60
α, β, γ (°)	90.0, 90.0, 90.0	90.0, 90.0, 120.0	90.0, 90.0, 120.0	90.0, 102.93, 90	90, 105.5, 90
Resolution (Å)	19.3-1.7 (1.78-1.70)	93.64-2.2 (2.29-2.20)	29-1.62 (1.62-1.66)	112.97-2.92 (3.10-2.92)	70.91-2.37 (2.37-2.43)
R_{meas}	9.6 (30.1)	10.3 (48.1)	11.4 (92.5)	9.4 (N/A)	17.7 (96.1)
$CC_{1/2}$ (%)	99.4 (91.2)	99.7 (96.3)	99.7 (59.9)	96.4(33.8)	100 (60)
$I / \sigma I$	8.9 (2.8)	12.6 (3.1)	11.1 (1.6)	24.4(1.4)	6.8 (1.2)
Completeness (%)	99.0 (98.8)	99.2 (98.5)	99.7 (95.6)	99.9 (98.8)	97.4 (74.4)
Redundancy	3.3 (3.1)	6.4 (5.6)	4.9 (3.7)	3.5 (3.4)	3.3 (2.4)
Resolution (Å)	1.7	2.2	1.62	2.92	2.37
No. reflections	17598	47982	24565	10246	15418
R_{work} / R_{free}	0.18/0.22	0.20/0.24	0.15/0.18	0.19/0.29	0.19/0.26
No. atoms	1657	5750	1619	2857	2875
Protein	1506	5614	1443	2815	2819
Ligand/ion	0	2	10	15	0
Water	151	135	152	24	56
B-factors					
Protein	21.81	41.62	17.99	57.69	30.68
Ligand/ion	0	46.39	33.52	92	N/A
Water	33.54	36.74	30.03	33.6	26.02
R.m.s. deviations					
Bond lengths (Å)	0.01	0.01	0.01	0.01	0.01
Bond angles (°)	1.60	1.40	1.7	1.9	1.7

*Values in parentheses are for highest-resolution shell.

Table S2 – ITC data. Concentration for proteins and peptides is given as monomer. * indicates where N was fixed in analysis. **Concentration of GYG1 (and MBP-T7-CC-PS) dimer was used in the analysis. NB = no binding.

TRIM7	CONC. [UM]	LIGAND	CONC. [UM]	SEQUENCE	N	K_D	ΔH (CAL/MOL)	TEMP[°C]
**HTR7-PS WT**	500	rbGYG1	100	see methods	1.07±0.014	2.2 µM±0.17	-7271±130	15
**MTR7-PS WT**	500	rbGYG1	100	see methods	0.93±0.005	1.5 µM±0.27	-1113±79	15
**MTR7-PS WT**	500	mGYG1	100	see methods	0.96±0.008	1.04 µM ±0.15	-1192±132	15
**HTR7-PS WT**	20	hGYG1-GN1	400	see methods	0.42±0.03 **	2.16 µM±0.06	-27000±3300	25
**HTR7-PS WT**	500	rbGYG1$_{VR-CIV}$	100	see methods	1.05±0.011	2.6 µM ±0.33	-6433±95	15
**HTR7-PS WT**	500	rbGYG1$_{CIV}$	100	see methods	0.70±0.034	4.8µM ±4.0	-9191±615	15
**HTR7-PS WT**	500	rbGYG$_{322-333}$	50	DNIKKKLDTYLQ	0.70±0.03	21 ± 1.8 µM	-9191±615	15
**HTR7-PS WT**	25	hGYG1$_{322-333}$	250	DNIKRKLDTYLQ	0.97±0.02	8 ± 0.64 µM	-11200±383	20
**HTR7-PS WT**	25	RACO-1$_{227-235}$	250	NLGLSMLQ	0.61±0.04	7.9 ± 1.06 µM	-6360±561	20
**HTR7-PS WT**	20	CVB-2C$_{208-214}$	400	TIEALFQ	0.90±0.2	11.2 ± 4.2 µM	-12600±3580	25
**HTR7-PS WT**	20	MNV1-NS6$_{1172-1178}$	400	LEALEFQ	1.13±1.62	8.7 ± 3.2 µM	-11700±2250	25
**HTR7-PS WT**	20	MNV1-NS3$_{700-706}$	400	HDDFGLQ	1.18±0.19	14.3 ± 4.3 µM	-10900±2280	25
**HTR7-PS WT**	20	polyA	400	AAAAAAA	NB	NB	NB	25
**HTR7-PS WT**	20	polyA-LQ	400	AAAAALQ	1*	8.92 ± 3.03 µM	-4870±513	25
**HTR7-PS WT**	20	LQ	2000	LQ	1*	201 ± 30 µM	-5720±507	25
**HTR7-PS WT**	20	Ac-LQ	800	Ac-LQ	1*	144 ± 26 µM	-6600±760	25
**HTR7-PS WT**	20	Ac-LLQ	800	Ac-LLQ	1*	38.7 ± 5.16 µM	-7720±484	25
**HTR7-PS WT**	20	TIEALFA	400	TIEALFA	NB	NB	NB	25
**HTR7-PS WT**	20	AIEALFQ	400	AIEALFQ	0.80±0.19	17.0 ± 4.3 µM	-15500±4410	25
**HTR7-PS N383A**	25	hGYG1$_{322-333}$	250	DNIKRKLDTYLQ	NB	NB	NB	20
**HTR7-PS T384A**	25	hGYG1$_{322-333}$	250	DNIKRKLDTYLQ	0.71±0.18	22.6 ± 6.6 µM	-7280±2410	20
**HTR7-PS R385A**	25	hGYG1$_{322-333}$	250	DNIKRKLDTYLQ	NB	NB	NB	20
**HTR7-PS L423A**	25	hGYG1$_{322-333}$	250	DNIKRKLDTYLQ	NB	NB	NB	20
**HTR7-PS F426A**	25	hGYG1$_{322-333}$	250	DNIKRKLDTYLQ	NB	NB	NB	20
**HTR7-PS Q436A**	25	hGYG1$_{322-333}$	250	DNIKRKLDTYLQ	NB	NB	NB	20
**HTR7-PS N438A**	25	hGYG1$_{322-333}$	250	DNIKRKLDTYLQ	0.64±0.02	2.32 ± 0.26 µM	-9830±318	20
**HTR7-PS N383A**	25	RACO-1$_{227-235}$	250	NLGLSMLQ	NB	NB	NB	20
**HTR7-PS T384A**	25	RACO-1$_{227-235}$	250	NLGLSMLQ	0.99±0.05	12.3 ± 2.00 µM	-2870±253	20
**HTR7-PS R385A**	25	RACO-1$_{227-235}$	250	NLGLSMLQ	NB	NB	NB	20
**HTR7-PS L423A**	25	RACO-1$_{227-235}$	250	NLGLSMLQ	NB	NB	NB	20
**HTR7-PS F426A**	25	RACO-1$_{227-235}$	250	NLGLSMLQ	NB	NB	NB	20
**HTR7-PS Q436A**	25	RACO-1$_{227-235}$	250	NLGLSMLQ	NB	NB	NB	20
**HTR7-PS N438A**	25	RACO-1$_{227-235}$	250	NLGLSMLQ	0.67±0.08	3.93 ± 1.75 µM	-4930±852	20
**HTR7-PS N383A**	20	CVB-2C$_{208-214}$	400	TIEALFQ	NB	NB	NB	25
**HTR7-PS T384A**	20	CVB-2C$_{208-214}$	400	TIEALFQ	1 *	21.2 ± 5.84 µM	-6120±704	25
**HTR7-PS R385A**	20	CVB-2C$_{208-214}$	400	TIEALFQ	NB	NB	NB	25
**HTR7-PS L423A**	20	CVB-2C$_{208-214}$	400	TIEALFQ	NB	NB	NB	25
**HTR7-PS F426A**	20	CVB-2C$_{208-214}$	400	TIEALFQ	NB	NB	NB	25
**HTR7-PS Q436A**	20	CVB-2C$_{208-214}$	400	TIEALFQ	NB	NB	NB	25
**HTR7-PS N438A**	20	CVB-2C$_{208-214}$	400	TIEALFQ	0.92±0.06	7.82 ± 1.18 µM	-10400±910	25
**HTR7-PS R385A**	20	MNV1-NS6$_{1172-1178}$	400	LEALEFQ	NB	NB	NB	25
**HTR7-PS R385A**	20	MNV1-NS3$_{700-706}$	400	HDDFGLQ	NB	NB	NB	25
**HTR7-CC-PS WT**	12	hGYG$_{322-333}$	300	DNIKRKLDTYLQ	1.7 ± 0.8	15 ± 6 µM	-9680±5770	25
**HTR7-CC-PS WT**	12	hGYG1-GN1	150	see methods	0.8 ± 0.02	0.16 ± 0.05 µM	-23800±991	25
